# Exploring new structural features of the 4-[(3-methyl-4-aryl-2,3-dihydro-1,3-thiazol-2-ylidene)amino]benzenesulphonamide scaffold for the inhibition of human carbonic anhydrases

**DOI:** 10.1080/14756366.2019.1654470

**Published:** 2019-08-20

**Authors:** Simona Distinto, Rita Meleddu, Francesco Ortuso, Filippo Cottiglia, Serenella Deplano, Lisa Sequeira, Claudia Melis, Benedetta Fois, Andrea Angeli, Clemente Capasso, Rossella Angius, Stefano Alcaro, Claudiu T. Supuran, Elias Maccioni

**Affiliations:** a Department of Life and Environmental Sciences, University of Cagliari, Cagliari, Italy;; b Dipartimento di Scienze della Salute, Università Magna Graecia di Catanzaro, Catanzaro, Italy;; c Dipartimento NEUROFARBA, Sezione di Scienze Farmaceutiche, Università degli Studi di Firenze, Sesto Fiorentino, Italy;; d Istituto di Bioscienze e Biorisorse, CNR, Napoli, Italy;; e Laboratorio NMR e Tecnologie Bioanalitiche, Pula, Italy

**Keywords:** Antitumour agents, carbonic anhydrase inhibitors, dihydrotiazoles, sulphonamide

## Abstract

A library of 4-[(3-methyl-4-aryl-2,3-dihydro-1,3-thiazol-2-ylidene)amino]benzene-1-sulphonamides (**EMAC8002a–m**) was designed and synthesised to evaluate the effect of substituents in the positions 3 and 4 of the dihydrothiazole ring on the inhibitory potency and selectivity toward human carbonic anhydrase isoforms I, II, IX, and XII. Most of the new compounds preferentially inhibit the isoforms II and XII. Both electronic and steric features on the aryl substituent in the position 4 of the dihydrothiazole ring concur to determine the overall biological activity of these new derivatives.

## Introduction

**Figure 1.**
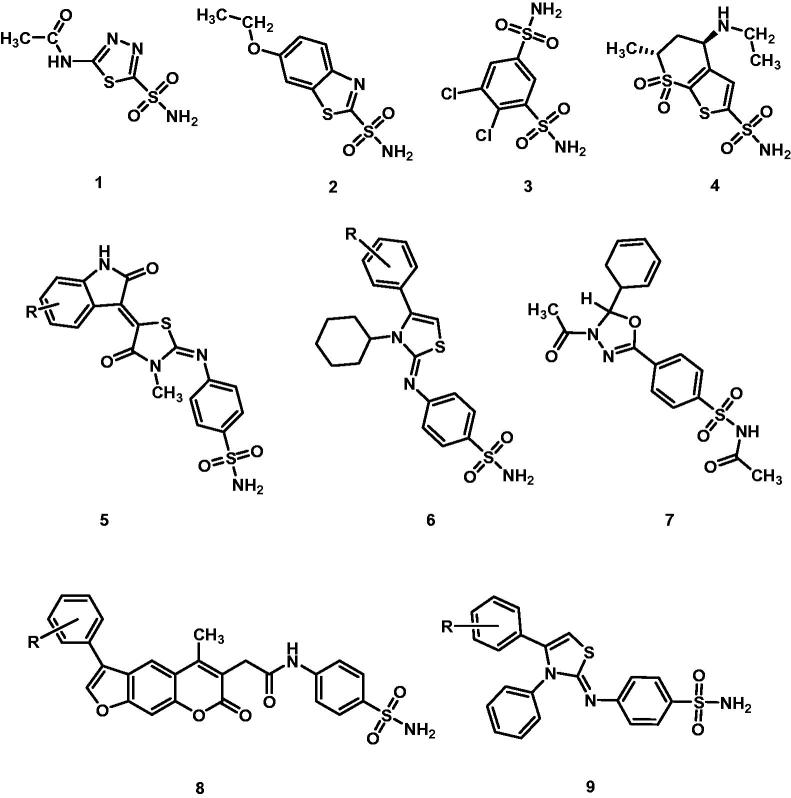
Carbonic anhydrase inhibitors in clinical use and previously reported EMAC derivatives: (1) acetazolamide (2) ethoxzolamide, (3) dichlorphenamide, (4) dorzolamide, (5) EMAC10020^47^, (6) EMAC8001^31^, (7) EMAC8000^48^, (8) EMAC10153^50^, (9) EMAC10111^49^.

1,3-thiazole and their hydrogenated analogues are important molecular subunits in diverse classes of biologically active molecules and thus can be found in several drugs approved for clinical use. Not surprisingly, this moiety has been extensively studied and both natural and synthetic thiazole derivatives are in therapeutic use or have shown potential therapeutic application toward several pathologies and targets such as bacteria^1^^,^^2^, tumours^3–6^, HIV-1 protease and reverse transcriptase^7–12^, fungi^13–15^, neurodegeneration and related pathologies^12^^,^^16^^,^^17^, and protozoal infections^18^. Recently, we reported on benzenesulphonamide dihydrothiazole derivatives as inhibitors of human carbonic anhydrase (hCA) isozymes I, II, IX, and XII19. This enzyme family catalyses the reversible hydration of carbon dioxide to bicarbonate and protons^19^ and, therefore, plays an essential role in CO_2_-related metabolism and in its transportation across biological membranes^20^^,^^21^. Due to their simple but essential role, hCAs have been recognised as main actors in a number of physiological processes and pathologies^22–30^. Not surprisingly, hCA inhibitors have been intensively studied and several are in clinical use for diverse pathologies^31–37^. Although different mechanisms of inhibition of hCA have been reported (e.g. coumarins, phenols, primary amines, COOMe derivatives)^38–42^, benzenesulphonamides and their isosters are the most represented molecular class of inhibitors^32^^,^^43–48^. These inhibitors belong to the so called zinc binders. They bind the zinc cofactor as conjugated bases and therefore, the acidity of the sulphonamide group influences their potency. Thus, by conjugating the benzenesulphonamide group to an electron withdrawing heterocyclic ring, the activity could be favourably influenced. Moreover, the introduction of further substituents in the heterocyclic core may influence the isozyme selectivity. On these bases, and according to our previous observation^31^^,^^47–50^, we have synthesised and evaluated for the inhibition activity toward hCA I, II, IX, and XII isoforms a series of the 4-[(3-methyl-4-aryl-2,3-dihydro-1,3-thiazol-2-ylidene)amino]benzenesulphonamides (Figure 1).

## Methods

### Materials and apparatus

Starting materials and reagents were obtained from commercial suppliers and were used without purification. All melting points were determined on a Stuart SMP11 melting points apparatus and are uncorrected. Electron ionisation mass spectra were obtained by a Fisons QMD 1000 mass spectrometer (Danvers, MA) (70 eV, 200 mA, ion source temperature 200 °C). Samples were directly introduced into the ion source. Melting points, yield of reactions, and analytical data of derivatives **EMAC8002a–l** are reported in Table 1.

**Table 1.** Chemical, analytical, and physical data of derivatives **EMAC8002 a–m**.


Compound	R	R.F.^a^	C–H–N		M.P. °C	Yield %	Mass fragments
			Calc.	Found			
**EMAC8002a**	4-Br	0.78	C, 45.29; H, 3.33; N, 9.90	C, 44.87; H, 3.28; N, 9.83	256-259	55	425; 423
**EMAC8002b**	4-Cl	0.70	C, 50.59; H, 3.71; N, 11.06	C, 50.61; H, 3.67; N, 11.02	241-242	76	379
**EMAC8002c**	4-F	0.63	C, 52.88; H, 3.88; N, 11.56	C, 52.90; H, 3.83; N, 11.51	230-233	58	363
**EMAC8002d**	3-NO_2_	0.74	C, 49.22; H, 3.61; N, 14.35;	C, 49.30; H, 3.58; N, 14.28;	235-236	73	390
**EMAC8002e**	2,4-Cl	0.83	C, 46.38; H, 3.16; N, 10.14	C, 46.42; H, 3.13; N, 10.09	256-257	79	413
**EMAC8002f**	4-CN	0.72	C, 55.12; H, 3.81; N, 15.12	C, 55.08; H, 3.77; N, 15.04	237-238	85	370
**EMAC8002g**	2,4-F	0.67	C, 50.38; H, 3.44; N, 11.02	C, 50.31; H, 3.40; N, 10.97	239-240	45	381
**EMAC8002h**	4-NO_2_	0.72	C, 49.22; H, 3.61; N, 14.35	C, 49.00; H, 3.58; N, 14.29	244-245	63	390
**EMAC8002i**	4-C_6_H_5_	0.76	C, 62.68; H, 4.54; N, 9.97	C, 62.68; H, 4.52; N, 9.98	254-255	72	421
**EMAC8002j**	4-CH_3_	0.77	C, 56.80; H, 4.77; N, 11.69	C, 56. 75; H, 4.80; N, 11.61	252-254	67	359
**EMAC8002k**	4-OCH_3_	0.65	C, 54.38; H, 4.56; N, 11.19	C, 54.37; H, 4.57; N, 11.17	243-244	77	375
**EMAC8002l**	//	0.69	C, 47.84; H, 3.73; N, 11.96	C, 47.80; H, 3.75; N, 11.89	233-235	74	351
**EMAC8002m**	H	0.74	C, 55.63; H, 4.38; N, 12.16	C, 55.55; H, 4.34; N, 12.13	252-253	67	345

a R.F. values were obtained on silica gel plates using a mixture of ethyl acetate/n-hexane 2/1.

^1^H-NMR (Table 2) were registered on a Bruker AMX (300 MHz) (chemical shifts in *δ* values) or on a Unity Inova 500NB high-resolution spectrometer (Agilent Technologies, CA) (500 MHz) All samples were measured in DMSO. Chemical shifts are reported referenced to the solvent in which they were measured. Coupling constants *J* are expressed in hertz (Hz). Elemental analyses were obtained on a Perkin–Elmer 240 B microanalyser. Analytical data of the synthesised compounds are in agreement within ± 0.4% of the theoretical values. TLC chromatography was performed using silica gel plates (Merck F 254), spots were visualised by UV light.

**Table 2.** 1H NMR data of derivatives **EMAC8002a–m**.

Compound	^1^H NMR δ (ppm)
**EMAC8002a**	^1^H-NMR: (300 MHz, DMSO) 3.57 (3H, s, CH_3_), 7.10 (1H, s, CH thiazole), 7.45 (2H, s, NH_2_, D_2_O), 7.54 (2H, d, *J* = 7.9 Hz, CH Ar), 7.61 (2H, d, *J* = 7.7 Hz, CH Ar), 7.78 (2H, d, *J* = 7.8 Hz, CH Ar), 7.95 (2H, d, *J* = 7.7 Hz, CH Ar)
**EMAC8002b**	^1^H-NMR: (300 MHz, DMSO) 3.46 (3H, s, CH_3_), 6.83 (1H, s, CH thiazole), 7.36 (2H, s, NH_2_, D_2_O), 7.44 (2H, d, *J* = 8.4 Hz, CH Ar), 7.58 (2H, d, *J* = 8.7 Hz, CH Ar), 7.63 (2H, d, *J* = 8.4 Hz, CH Ar), 7.88 (2H, d, *J* = 8.5 Hz, CH Ar)
**EMAC8002c**	^1^H-NMR: (300 MHz, DMSO) 3.65 (3H, s, CH_3_), 7.01 (1H, s, CH thiazole), 7.50 (2H, t, *J* = 8.5 Hz, CH Ar), 7.56 (2H, s, NH_2_, D_2_O), 7.67 (2H, d, *J*: 8.7 CH Ar), 7.74 (2H, dd, *J*H-H: 8.5, *J*H-F: 5.5, CH Ar), 8.02 (2H, d, *J*: 9.0, CH Ar)
**EMAC8002d**	^1^H-NMR: (300 MHz, DMSO) NH_2_ not detected, 3.34 (3H, s, CH_3_), 6.59 (1H, s, CH thiazole), 7.18 (2H, d, *J* = 8.5 Hz, CH Ar), 7.24 (1H, s, CH Ar), 7.78 (2H, d, *J* = 8.5 Hz, CH Ar), 7.83 (1H, d, *J* = 7.8 Hz, CH Ar), 8.01 (1H, d, *J* = 7.8 Hz, CH Ar), 8.34 (1H, d, *J* = 2.0 Hz, CH Ar)
**EMAC8002e**	^1^H-NMR: (500 MHz, DMSO) 3.25 (3H, s, CH_3_), 6.68 (1H, s, CH thiazole), 7.29 (2H, s, NH_2_, D_2_O), 7.34 (2H, d, *J* = 8 Hz, CH Ar), 7.59 (1H, d, *J* = 8 Hz, CH Ar), 7.63 (1H, dd, J = 8.5, 2 Hz), 7.83 (2H, d, *J* = 8.5 Hz, CH Ar), 7.88 (1H, d, *J* = 2 Hz, CH Ar)
**EMAC8002f**	^1^H-NMR: (300 MHz, DMSO) 3.46 (3H, s, CH_3_), 6.83 (1H, s, CH thiazole), 7.36 (2H, s, NH_2_, D_2_O), 7.44 (2H, d, *J* = 8.4 Hz, CH Ar), 7.58 (2H, d, *J* = 8.7 Hz, CH Ar), 7.63 (2H, dd, *J* = 8.4 Hz, CH Ar), 7.88 (2H, d, *J* = 8.5 Hz, CH Ar)
**EMAC8002g**	^1^H-NMR: (300 MHz, DMSO) 3.55 (3H, s, CH_3_), 7.13 (1H, s, CH thiazole), 7.34 (1H, td, *J* = 8.4, 1.5 Hz, CH Ar), 7.45 (2H, s, NH_2_, D_2_O), 7.67-7.53 (4H, m, CH Ar), 7.93 (2H, d, *J* = 7.6 Hz, CH Ar)
**EMAC8002h**	1H-NMR: (300 MHz, DMSO) 3.36 (3H, s, CH_3_), 6.61 (1H, s, CH thiazole), 7.17 (2H, d, *J* = 8.2 Hz, CH Ar), 7.25 (2H, s, NH_2_, D_2_O), 7.79 (2H, d, *J* = 8.5 Hz, CH Ar), 7.83 (2H, d, *J* = 8.7 Hz, CH Ar), 8.33 (2H, d, *J* = 8.0 Hz, CH Ar)
**EMAC8002i**	^1^H-NMR: (500 MHz, DMSO) 3.59 (3H, s, CH_3_), 7.0 (1H, s, CH thiazole), 7.40 (2H, s, NH_2_)_,_ 7.43 (1H, m, CH Ar), 7.52 (2H, m, CH Ar), 7.56 (2H, d, J = 8 Hz, CH Ar), 7.66 (2H, d, *J* = 8 Hz, CH Ar), 7.75 (2H, m,CH Ar), 7.87 (2H, d, *J* = 8.5 Hz, CH Ar), 7.93 (2H, d, J = 8.5 Hz)
**EMAC8002j**	^1^H-NMR: (300 MHz, DMSO) 2.40 (3H, s, CH_3_), 3.53 (3H, s, CH_3_), 6.91 (1H, s, CH thiazole), 7.46-7.36 (6H, m, CH Ar), 7.55 (2H, s, NH_2_, D_2_O), 7.92 (2H, d, *J* = 8.5 Hz, CH Ar)
**EMAC8002k**	^1^H-NMR: (300 MHz, DMSO) 3.64 (3H, s, CH_3_), 3.96 (3H, s, OCH_3_), 6.98 (1H, s, CH thiazole), 7.23 (2H, d, *J* = 8.5 Hz, CH Ar), 7.54 (2H, s, NH_2_, D_2_O), 7.63-7.60 (4H, m, CH Ar), 8.04 (2H, d, *J* = 8.1 Hz, CH Ar)
**EMAC8002l**	^1^H-NMR: (300 MHz, DMSO) 3.61 (3H, s, CH_3_), 7.15 (1H, s, CH thiazole), 7.48 (2H, s, NH_2_, D_2_O), 7.59 (5H, m, CH Ar), 7.67 (2H, d, *J* = 8.5 Hz, CH Ar), 7.97 (2H, d, *J* = 8.5 Hz, CH Ar)
**EMAC8002m**	^1^H-NMR: (300 MHz, DMSO) 3.61 (3H, s, CH_3_), 7.15 (1H, s, CH thiazole), 7.48 (2H, s, NH_2_, D_2_O), 7.59 (5H, m, CH Ar), 7.67 (2H, d, *J* = 8.5 Hz, CH Ar), 7.97 (2H, d, *J* = 8.5 Hz, CH Ar)

## General procedure for the synthesis of compound EMAC8002a–m

### Synthesis of 1-methyl-3–(4-sulfamoylethyl)thiourea

To an ethanolic solution of 4-aminobenzenesulphonamide (1 eq), methyl isothiocyanate (2 eq) was added dropwise. The mixture was heated under reflux until the completion of the reaction (10 h). The progress of the reaction was monitored by TLC (ethyl acetate/n-hexane 2/1). Then the reaction was cooled overnight in the fridge. A precipitate was formed which was collected by filtration under vacuum and crystallised from ethanol to afford the desired product.

### Synthesis of 4-[(3-methyl-4-aryl-2,3-dihydro-1,3-thiazol-2-ylidene)amino]benzenesulphonamide

A mixture of 1-methyl-3–(4-sulfamoylphenyl)thiourea (1 eq) and α-halogenoketone (1 eq) was reacted in ethanol solution. Different reaction conditions have been employed. Thus, while in the presence of α-bromoketones the reaction temperature was kept between 30 and 50 °C, refluxing conditions were used when α-chloroketones were reacted. The mixture was reacted until completion (TLC, ethyl acetate/n-hexane 2/1). By cooling to room temperature, a precipitate was formed. The crude product was filtered and crystallised from the appropriate solvent. Analytical and spectral data of compounds **EMAC8002a–m** are reported in Tables 1 and 2.

## Molecular modelling

The new ligand **EMAC8002i** was built by means of Maestro GUI^51^ in E configuration. Then a conformational search analysis was performed using MCMM method allowing 5000 iterations in implicit solvent^52^.

Docking experiments were performed by means of Glide Quantum-Mechanical Polarised Docking^53^^,^^54^. The crystallographic model with the best resolution was considered (pdb code 5MSA, 1.2 Å). The protein was prepared with Preparation Wizard protocol. The Grid box was centred on the co-crystallised ligand and all parameters were set up as default.

The best pose complex was then minimised to consider the induced fit phenomena and used to analyse the ligand-binding mode. 10,000 steps of the Polak-Ribier conjugate gradient (PRCG) minimisation method were conducted on the top ranked theoretical complex using OPLS_2005 force field^55^.

The optimisation process was performed up to the derivative convergence criterion equal to 0.05 kcal/(mol*Å)^−1^.

## Biological activity

### Carbonic anhydrase inhibition assay

The purification of cytosolic CA isoenzymes (CA I and CA II) was previously described with a simple one-step method by a Sepharose-4B-L tyrosine-sulphanilamide affinity chromatography^56^.

The protein quantity in the column effluents was determined spectrophotometrically at 280 nm. Sodium dodecyl sulphate polyacrylamide gel electrophoresis (SDS-PAGE) was applied with a Bio-Rad Mini Gel system Mini-PROTEINVR system (Hercules, CA), Bio-Rad Laboratories, Inc., China after purification of both CA isoenzymes. Briefly, it was performed in acrylamide for the running (10%) and the stacking gel (3%) contained SDS (0.1%), respectively. Activities of CA isoenzymes were determined according to a method by Verporte et al.^57^. The increase in absorbance of the reaction medium was spectrophotometrically recorded at 348 nm. Also, the quantity of protein was determined at 595 nm according to the Bradford method^58^. Bovine serum albumin was used as standard protein. The IC_50_ values were obtained from activity (%) versus compounds plots^59^. For calculation of *K*_I_ values, three different concentrations were used. The Lineweaver–Burk curves were drawn and calculations were realised^59^. The biological data are reported in Table 3.

**Table 3.** Inhibition data towards hCA I, II, IX, and XII of compounds **EMAC8002 a–m**.


		*K*_i_ (nM)			
Compound	R	hCA I	hCA II	hCA IX	hCA XII
**EMAC8002a**	4-Br	4457	45.8	58.1	778
**EMAC8002b**	4-Cl	1548	13.1	1213	97.9
**EMAC8002c**	4-F	4845	5.3	2482	40.0
**EMAC8002d**	3-NO_2_	2456	17.8	1821	271
**EMAC8002e**	2,4-Cl	>10,000	398	1605	10.5
**EMAC8002f**	4-CN	2650	17.9	2064	94.7
**EMAC8002g**	2,4-F	>10,000	37.7	1648	9.0
**EMAC8002h**	4-NO_2_	2239	19.9	142	9.5
**EMAC8002i**	4-C_6_H_5_	8910	503	1389	9.5
**EMAC8002j**	4-CH_3_	2603	4.5	23.3	3.1
**EMAC8002k**	4-OCH_3_	5580	3.8	25.4	4.6
**EMAC8002l**	//	1650	30.3	1874	36.9
**EMAC8002m**	H	2305	5.3	620	9.4
AAZ	//	250	12	25	5.7

## Results and discussion

As a continuation of our ongoing research in the field of carbonic anhydrase and anticancer agents^31^^,^^47–49^^,^^60^, we have synthesised a new series of 4-[(3-methyl-4-aryl-2,3-dihydro-1,3-thiazol-2-ylidene)amino]benzenesulphonamides, namely compounds **EMAC8002a–m**, to evaluate their activity and selectivity toward CA isozymes and to gain information on the structure–activity relationships of these derivatives. All the synthesised derivatives are characterised by the presence of a benzenesulphonamide moiety as zinc binder group, conjugated with the position 2 of a dihydrothiazole heterocyclic core. A methyl substituent is always present on the heterocyclic nitrogen atom, while a differently substituted aromatic ring occupies the position 4.

**Scheme 1.**
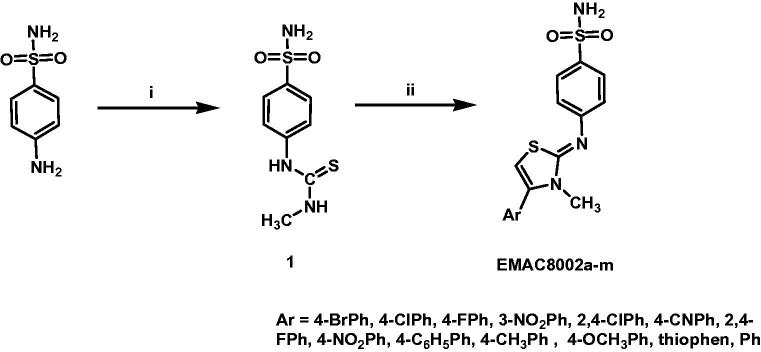
Synthetic pathway to compounds **EMAC8002a–m**. Reagents and conditions: (i) ethanol, methylisothiocyanate; (ii) ethanol, α-halogenoarylketone.

The synthetic procedure to obtain compounds **EMAC8002a–m** is depicted in Scheme 1. Briefly, it consists of two steps: the synthesis of the 3‐methyl‐1‐(4‐sulfamoylphenyl)thiourea (1) by reaction of the 4-aminobenzensufonamide with methyl isocyanate. The second step is the formation of the 4-aryl dihydrothiazole nucleus. It was accomplished by reacting 1 with the appropriate α-halogenoketone in ethanol solution. **EMAC8002a–m** were characterised by means of analytical and spectroscopic methods (Tables 1 and 2) and then submitted to enzymatic evaluation toward hCA I, II, IX, and XII.

The results are summarised in Table 3. Accordingly with our previous observations with similar derivatives, none of the **EMAC8002** compounds was active toward hCA I isozyme. On the contrary, when isozymes II, IX, and XII are investigated, some consideration regarding the structure–activity relationships could be done. When compounds **EMAC8002** are tested on hCA II, the introduction of a halogen atom, in position 4 of the phenyl moiety, in position 4 of the dihydrothiazole ring, appeared beneficial for the activity. However, the larger is the atomic radius of the halogen, the lower the activity. So far, the *K*_i_ values are in the following order: 4-F (**EMAC8002c**) 5.3 nM <4-Cl (**EMAC8002b**) 13.1 nM <4-Br (**EMAC8002a**) 45.8 nM. A similar behaviour was observed in the case of hCAXII: 4-F (**EMAC8002c**) 5.3 nM <4-Cl (**EMAC8002b**) 13.1 nM <4-Br (**EMAC8002a**) 45.8 nM. On the contrary, when the same compounds were evaluated on hCA IX, a totally reversed trend was observed. In fact, the larger is the halogen atom the higher the activity. Accordingly, the *K*_i_ values are 4-F (**EMAC8002c**) 2482 nM > 4-Cl (**EMAC8002b**) 1213 nM > 4-Br (**EMAC8002a**) 58.1 nM. The introduction of a second halogen atom in the position 2 of the phenyl ring is beneficial only for the activity toward the hCA XII isoform. Thus, in the case of compounds **EMA8002e** (2,4-Cl) and **EMAC8002g** (2,4-F) the Ki values are 10.5 and 9.0 nM, respectively. Accordingly, when the 4-Cl derivative (**EMAC8002b)** is compared with the 2,4-diCl one (**EMAC8002e),** a 9-fold gain in potency toward the hCA XII isozyme was observed. Similarly, a 4-fold increase in potency toward hCA XII was observed when 4-F (**EMAC8002c**) and 2,4-diF (**EMAC8002g**) are compared. On the contrary, the inverse was observed when hCA II isozyme is considered. A decrease in the activity of 7 folds was observed when 2,4-diF (**EMAC8002g**) was compared with 4-F (**EMAC8002c**). Analogously a decrease of the inhibition potency of 30 folds was measured when 2,4-diCl (**EMAC8002e**) was compared with 4-Cl (**EMAC8002b**). On these bases, we can summarise that by introducing specific halogen atoms in specific positions of the 4-phenyl ring, it is possible to modulate the activity toward different hCA isozymes. The introduction of a nitro group is tolerated both in the 3 and 4 position of the phenyl ring when hCA II is considered. On the contrary, when the activity on hCA XII is measured, the introduction of the nitro group is only tolerated in the 4 position. The introduction of electron-donating groups such as methyl or methoxy, as in compounds **EMAC8002j** and **EMAC8002k**, respectively, led to most active compounds when hCA II, IX, and XII are considered. Unfortunately, none of the two substitutions led to selective compounds. The presence of a nitrile in the position 4 as in the case of compound **EMAC8002f**, is generally detrimental for the activity, but for hCAII, where this substitution is tolerated. By introducing a biphenyl group in the position 4 of the dihydrothiazole, the most selective compound toward hCA XII (**EMAC8002i**) was obtained, with a selective index hCA II/hCA XII higher than 52. When compared with 4-phenyldihydrothiazole (**EMAC8002m**), the isosteric introduction of a thiophene-2-yl moiety in the position 4 of the dihydrothiazole (**EMAC8002l**) was beneficial only for the activity toward hCA I, although with high values of Ki.

These results, together with our previous findings, indicate that the 4-[(3-methyl-4-aryl-2,3-dihydro-1,3-thiazol-2-ylidene)amino]benzenesulphonamide scaffold could be rationally and efficiently decorated in order to achieve potent and selective hCA inhibitors.

**Figure 2.**
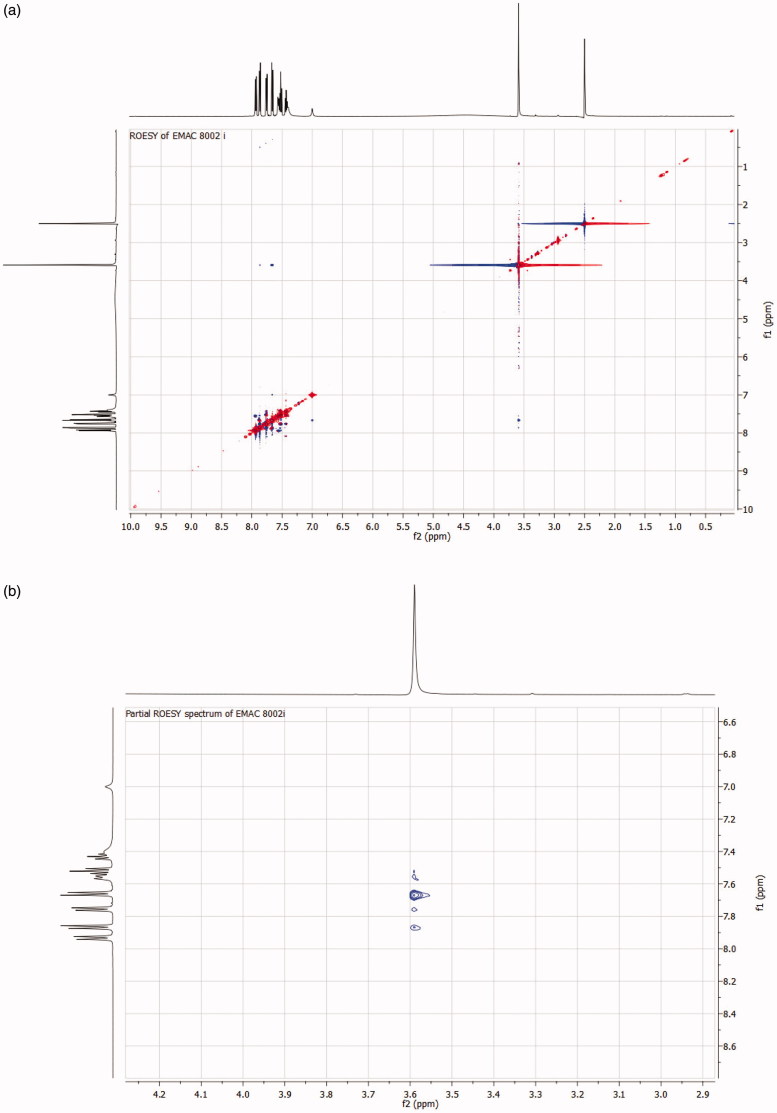
(a) ROESY spectrum of compound **EMAC8002i**; (b) Partial ROESY spectrum of compound **EMAC8002i**.

The possible formation of both E and Z diastereoisomers along the C=N double bond was investigated by 2D NMR experiments. To this end, the ROESY spectrum of the most interesting compound of the series **EMAC8002i** was recorded (Figure 2). This derivative showed good selectivity toward hCA XII and inhibitory activity toward this enzyme in the low nM range. ROESY cross-peak from the methyl group at δ_H_ 3.59 (3H, s) to the aromatic protons H-2 and H-3 at δ_H_ 7.56 (2H, *d*, *J* = 8 Hz) permitted to assign the configuration around the double bond as E. In fact, examination of a molecular model confirms that, in the case of (Z) configuration, H-2 and H-3 aromatic protons would be too far to the methyl group and correlation should not be observed. Analogous experiments were performed along the full series of compounds and, as expected, the (E) configuration was assigned to all derivatives.

**Figure 3.**
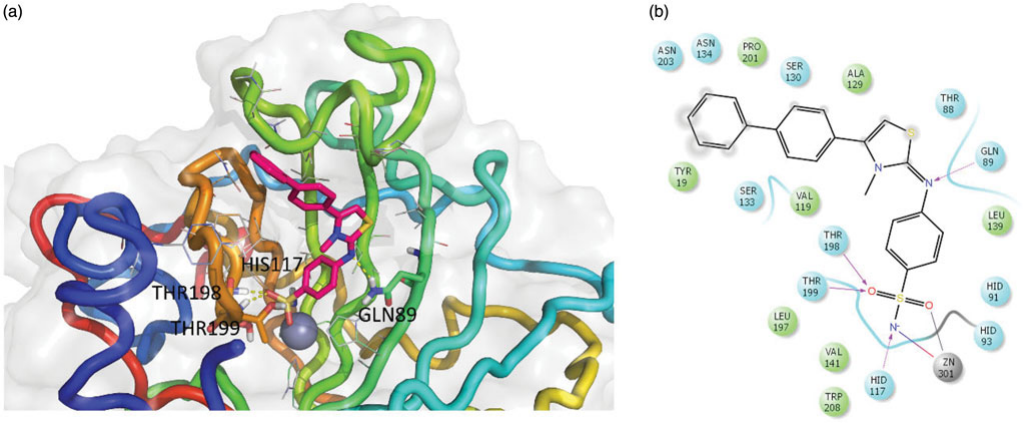
Three-dimensional representation of the putative binding mode as obtained by docking experiments of: (a) **EMAC8002-i** and (b) relative 2D representation of the complex stabilising interactions with the residues of the binding site.

In order to predict the binding mode of compound **EMAC8002i**, a molecular docking experiment was performed. The most selective was well docked into the catalytic site of CA XII with binding energies of −10.194 kcal/mol. The complex has been energy minimised and the putative binding mode is depicted in Figure 3.

The ligand well fit into the binding pocket. The benzenesulphonamide moiety tightly interacts with the deep cavity and the Zn (II) ion, being stabilised by the metal chelation and an array of hydrogen bonds with the residues around the ion: Thr198, Thr199, and His117. Furthermore, the nitrogen atom of the aminobenzenesulphonamide moiety interacts with Gln89. Moreover, the substituent in the position 4 of the thiazolino portion interacts with the external part of the cavity. The analysis of the putative binding mode highlighted the presence of extra space in correspondence of the N methyl substituent which will be exploited to increase the ligand complementarity and probably its activity and selectivity.

## Conclusions

We have designed and synthesised a series of 4-[(3-methyl-4-aryl-2,3-dihydro-1,3-thiazol-2-ylidene)amino]benzenesulphonamides and evaluate their activity on hCA I, II, IX, and XII isozymes. While these derivatives were weak inhibitors of the hCA I isoform, interestingly, the nature and substitution pattern of the aromatic group in the position 4 of the dihydrothiazole core was relevant for the activity and the selectivity between the hCA II and XII isoforms. Nevertheless, we observed that the introduction of a 4-methylphenyl or a 4-methoxyphenyl moiety in the position 4 of the dihydrothiazole ring is beneficial for the activity toward hCA II, IX, and XII isozymes. These data prompted us to further investigate on these scaffolds in order to optimise both the activity and the isozyme selectivity.
